# Rumen protozoa are a hub for diverse hydrogenotrophic functions

**DOI:** 10.1111/1758-2229.13298

**Published:** 2024-07-03

**Authors:** Ido Toyber, Raghawendra Kumar, Elie Jami

**Affiliations:** ^1^ Department of Ruminant Science, Institute of Animal Sciences Agricultural Research Organization, Volcani Center Rishon LeZion Israel; ^2^ Department of Animal Science the Hebrew University of Jerusalem Rehovot Israel

## Abstract

Ciliate protozoa are an integral part of the rumen microbial community involved in a variety of metabolic processes. These processes are thought to be in part the outcome of interactions with their associated prokaryotic community. For example, methane production is enhanced through interspecies hydrogen transfer between protozoa and archaea. We hypothesize that ciliate protozoa are host to a stable prokaryotic community dictated by specific functions they carry. Here, we modify the microbial community by varying the forage‐to‐concentrate ratios and show that, despite major changes in the prokaryotic community, several taxa remain stably associated with ciliate protozoa. By quantifying genes belonging to various known reduction pathways in the rumen, we find that the bacterial community associated with protozoa is enriched in genes belonging to hydrogen utilization pathways and that these genes correspond to the same taxonomic affiliations seen enriched in protozoa. Our results show that ciliate protozoa in the rumen may serve as a hub for various hydrogenotrophic functions and a better understanding of the processes driven by different protozoa may unveil the potential role of ciliates in shaping rumen metabolism.

## Graphical abstract

Our study investigates the role of ciliate protozoa in the rumen microbial community, focusing on their stable association with specific prokaryotic taxa and their involvement in hydrogenotrophic functions. By altering forage to concentrate ratios, we induce major changes in the prokaryotic community, yet find that certain taxa persistently associate with ciliate protozoa. Our findings suggest that ciliate protozoa act as hubs for hydrogen utilization pathways, offering insights into their potential role in shaping rumen metabolism.

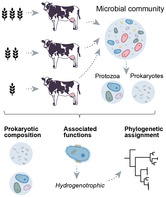

## INTRODUCTION

The rumen microbial community is considered to be one of the most diverse and complex among host‐associated communities (de Jonge et al., 2022). Unlike monogastric animals in which microbial fermentation represents but a fraction of the total energy balance of the host, the rumen microbiome is responsible for the vast majority of the carbon and nitrogen requirements of the ruminant animal (Bergman, 1990). This is performed by a vast microbial community encompassing all domains of life; bacteria, archaea and microbial eukaryotes. The feed ingested by the host is broken down by the microbes involving complex cascades of cross‐feeding and interactions that lead to the production of end products for the animal in the form of volatile fatty acids as well as the production of the unusable methane. Although the understanding of the rumen microbial ecology as well as the role and interactions between the rumen bacterial community has increasingly been studied, the eukaryotic portion of the rumen microbiome, their dynamics in the rumen and interactions remain largely neglected (Firkins et al., 2020; Mizrahi & Jami, 2021; Solomon & Jami, 2021).

The largest fraction of the rumen eukaryotic community is the ciliate protozoa, which account for 2% of all microbial species and can account for up to 50% of the biomass (Cottle et al., 1978; Newbold et al., 2015). Several studies, such as Henderson et al, surveyed ruminant species across different geographies and diets, emphasized that ciliate protozoa may be more diverse than originally expected and that they may not be constrained by the same ecological forces as the prokaryotic community (Cui et al., 2022; Henderson et al., 2015).

In addition to their still elusive ecological dynamics, ciliate protozoa have also been suggested to have many metabolic and physiological functions in the rumen, most of which have not yet been elucidated (Li et al., 2022). Some of these functions are further suggested to be a direct consequence of their interactions with the prokaryotic community (Ushida et al., 1997; Villar et al., 2020). The most studied functional interaction is their role in enhancing methane production via interspecies hydrogen transfer to methanogenic archaea (Ushida et al., 1997). Such interaction was observed across a wide range of in‐vivo and in‐vitro studies, with the estimated enhancement of methane due to the presence of protozoa ranging from 11% to 37% (Newbold et al., 2015; Ranilla et al., 2007; Solomon et al., 2021; Ushida et al., 1997), and not limited to the rumen environment (Treitli et al., 2023; Yamada et al., 1997). Additional evidence for the interaction stems from the physical association observed between ciliate protozoa and methanogens (Levy & Jami, 2018; Lloyd et al., 1996; Park & Yu, 2018). These studies note that, in addition to methanogens, protozoa likely harbour a diverse bacterial community for which as of yet no type of interaction has been proposed.

In this study, we investigate the effect of host diet on the protozoa composition and the composition of their associated prokaryotic community. We find that the protozoa community is less affected by dietary changes compared to the prokaryotic community. We also discover that while the protozoa‐associated prokaryotic community is dependent on the free‐living (FL) community and thus changes with diets, the protozoa microenvironment retains a unique community of enriched taxa independently of their abundance in the FL community. The enrichment of these taxa may be the result of functional interactions similar to methanogens, driven by their production of hydrogen.

## EXPERIMENTAL PROCEDURES

### Animals handling and sampling

The experimental procedures used in this study were approved by the Faculty Animal Policy and Welfare Committee of the Agricultural Research Organization Volcani Research Center approval no. 889/20 IL, in accordance with the guidelines of the Israel Council for Animal Care.

For the experiment, 15 cows, that were fed prior to the beginning of the experiment a high fibre diet, were divided into three groups by their fibre/grain ratio: Low‐Fibre (LF) refers to a total mixed ration (TMR) composed of ~70:30 grain to fibre ratio, Medium‐Fibre (MF) refers a feed ration composed of a grain to fibre ratio 50:50, and High‐Fibre (HF) refers to the cows receiving a diet composed of 20:80 grain to fibre ratio. The animals were maintained on their respective diet for 2 months prior to sampling.

### Rumen sample processing

Rumen fluid was collected via a custom‐made stainless‐steel stomach tube, connected to an electric‐powered vacuum pump (Gast, Inc., MI, USA). All samplings were performed 2 h after morning feeding. Whole rumen fluid was used for the counting and characterization of the protozoa community, with a subset of the sample centrifuged at 500 × *g* for 5 min at 4°C to obtain the FL prokaryotic community. The lack of protozoa in the prokaryotic community was validated by visual observation under the microscope.

For the isolation of the protozoa‐associated prokaryotic community, the remainder of the rumen fluid sampled (~1 L) was squeezed through eight layers of cheesecloth to remove plant particles, and dispensed into CO_2_‐filled glass bottles and immediately transferred to an oxygen‐free environment in an anaerobic glove box (Coy Inc., MI, USA). In the anaerobic glove box, the rumen fluid was transferred to a separating funnel to perform sedimentation of the protozoa as previously performed (Belanche et al., 2014; Levy & Jami, 2018; Williams & Coleman, 2012). Briefly, under an anaerobic environment, the rumen fluid was mixed at 1:1 ratio with pre‐warmed, anaerobic Coleman salt buffer and incubated for 50 min at 39°C (Williams & Coleman, 2012). Afterward, glucose was added in 1 g/L concentration to enhance flocculation of plant material for 20 min (Williams & Coleman, 2012). To clean the protozoa fraction from external prokaryotic cells stemming from the FL population, the protozoa community was washed four times by centrifugation at 500 × *g* for 5 min and subsequent fresh buffer replacement. The protozoa pellet obtained from the last washing step was suspended in a 3 mL extraction buffer and immediately frozen and kept at −20°C for downstream processing and analysis. As performed in a previous study, the buffer from the last washes was assessed for contamination with external prokaryotic cells by extracting DNA and amplification of the 16S rRNA gene to confirm the lack of FL bacteria in the protozoa‐associated samples (Levy & Jami, 2018).

### Protozoa visual quantification

To determine the number of protozoa in the whole rumen samples we took 1 mL of whole rumen fluid and centrifuged it at 500 × *g* for 5 min. Next, we suspended the protozoa pellet with a 1:10 ratio of 4% PFA at 7.4 pH and added two drops of brilliant green dye for protozoa staining and incubated for 1 h for better staining results. The fixed 1 mL protozoa suspension was transferred to a Sedgewick Rafter counting chamber (Marienfeld, Germany), and counted the number of protozoa on each grid under an 10× microscope objective lens (Zeiss, Germany). Overall for each sample, at least 25 squares were counted and the number of protozoa was extrapolated to the number of protozoa in 1 mL of rumen.

### DNA extraction

DNA extraction for the samples was performed as previously described (Stevenson & Weimer, 2007). In brief, cells were lysed by bead disruption using a Biospec Mini‐Beadbeater‐16 (Biospec, Bartlesville, OK, USA) at 3000 RPM for 3 min with phenol followed by phenol/chloroform DNA extraction. The final supernatant was precipitated with 0.6 volume of isopropanol and resuspended overnight in 50–100 μL TE buffer (10 mM Tris–HCl, 1 mM EDTA), then stored at 4°C for short‐term use, or archived at −80°C.

### Illumina amplicon sequencing

Amplicon sequencing was performed by the Hylab Research Laboratory (Rehovot, Israel). Briefly, 20 ng of DNA was used in a 25 μL PCR reaction with primers, using PrimeStar Max DNA Polymerase (Takara) for 20 cycles. The PCR product was purified using AmpureXP beads, and a second PCR to add the adapter and index sequences was performed using the Fluidigm Access Array primers for Illumina. The PCR reaction was purified using AmpureXP beads and the concentrations were measured by Qubit. The samples were pooled, run on a DNA D1000 screen tape (Agilent) to check for correct size and for the absence of primer‐dimers product. The pool was then sequenced on the Illumina MiSeq, using the MiSeq V2‐500 cycles sequencing kit.

Sequencing was performed on a MiSeq platform using the paired end protocol (2 × 250 bp). The primers sequences used for the 16S rRNA bacteria and archaea were taken from the updated sequences of the earth microbiome project, amplifying the V4 region 515F (5′‐GTGYCAGCMGCCGCGGTAA‐3′) and 806R (5′‐GGACTACNVGGGTWTCTAAT‐3′) (Caporaso et al., 2011). For the 18S rRNA sequencing, primers specifically designed for ciliates were used from Tapio et al. (2016) with the following sequences: CiliF (5′‐CGATGGTAGTGTATTGGAC‐3′) and CiliR (5′‐GGAGCTGGAATTACCGC‐3′) (Tapio et al., 2016).

### Quantitative real‐time PCR for microbial domains and key genes in hydrogenotrophic pathways

To determine the copy number of the different ruminal microbial populations and the bacterial hydrogenotrophic genes, we use the standard curve method (Yuan et al., 2006). The curve for each sequence was obtained by first amplifying sequences of our targeted genes; the PCR products were run and extracted from a 1.5% agarose gel. Each amplification product was quantified for the number of copies and serial 10‐fold dilution was made. Real‐time PCR was performed in a 10 μL reaction mixture containing 5 μL of Absolute Blue SYBR Green Master Mix (Thermo Scientific, Waltham, MA, United States), 0.5 μL of each primer (10 μM working concentration), 2 μL of nuclease‐free water and 2 μL of 10 ng/μl DNA templates. The genes tested include the formyltetrahydrofolate synthetase genes and acetyl‐COA synthase (*ftfhs*, *acsB*) of the Wood Ljungdahl pathway (Gagen et al., 2010), the adenosine‐5′‐phosphosulfate reductase (*aprA*) and the dissimilatory sulfite reductase subunit alpha (*dsrA*) (Devkota et al., 2012; Meyer & Kuever, 2007) of the dissimilatory sulfate reduction pathway and methyl coenzyme‐M reductase (*mcrA*) for hydrogenotrophic methanogenesis (Denman et al., 2007). For the ammonia forming nitrite reductase (*nrfA*) a primer set was designed in‐house by aligning the gene sequences from a dereplicated rumen MAGs in‐house database stemming from (Stewart et al., 2019; Xie et al., 2021). The NCBI accession numbers for the MAGs used for the primer design include CADAOT01 and CADBKQ01 Desulfovibrio_piger_ATCC_29098, RGIG1737, RGIG1974, RGIG3713, RGIG5510, RGIG6612, RGIG7164 and CADCDR01. The primers used throughout this experiment and the conditions for the reactions can be found in Table S1.

### Clone library, sequence alignment, phylogenetic tree, and taxonomic annotation

The PCR products of the representative genes of the hydrogenotrophic pathways were run on 1.5% agarose gel and purified using Gel DNA Recovery Kit (Hylabs, Rehovot, Israel), cloned in the PGEM‐T easy vector (Promega, USA) and transformed into competent *Escherichia coli* cells (DH5α). At least 10 clones per gene were picked for Sanger sequencing in order to assess the phylogenetic and taxonomic affiliation of the genes. For this analysis, we used previously performed large‐scale metagenomic studies with thousands of metagenome‐assembled genomes (MAGs), and compared the extracted sequences to the ones obtained in this study (Stewart et al., 2019; Xie et al., 2021). We additionally used Blast using the nr database on our sequences to uncover sequences not covered by the mentioned databases. We additionally added a comparison with sequences stemming from bacterial isolates known to carry a given pathway. All the sequences were trimmed based on the primer coverage and aligned using MAFFT (version 7) (Katoh et al., 2019). CD‐hit was used to remove redundancies in sequences at 97% similarity (except for the sequences obtained from this study) (Fu et al., 2012). The resulting alignment was used for the construction of a maximum‐likelihood phylogenetic tree using IQTree (Minh et al., 2020), with LG model and 1000 bootstrap replicates. The phylogenetic tree was visualized using iTOL (Letunic & Bork, 2019). We further assessed the complementarity of the pathways for each existing genome from bacterial isolates and MAGs from large‐scale metagenomic analyses performed on the rumen.

We further assessed the complementarity of the pathways for each existing genome, and MAG used the Prokka annotation of the genome (Seemann, 2014). Afterward, each genome protein annotated file was uploaded to the web‐based tool KofamKOALA–KEGG Orthology Search and each KEGG hit was mapped in the KEGG map with the default threshold value and searched for pathway completeness (Aramaki et al., 2020; Kanehisa & Goto, 2000).

### Data analysis

Downstream processing of the 16S rRNA data, up to the generation of the amplicon sequence variant table (ASV) was performed in QIIME v.2 (Bolyen et al., 2019). DADA2 was applied to model and correct Illumina‐sequencing amplicon errors and clustering of ASVs (Callahan et al., 2016). Taxonomic assignment for the bacterial 16S rRNA was performed using the pre‐trained classifier Silva database (Silva_138.1_ssu) ASVs from 515F/806R region from QIIME v.2 pipeline (Quast et al., 2013). For a more accurate and rumen specific taxonomic assignment of the archaeal sequences, the Rumen and Intestinal Methanogen database (RIM‐DB), which enables a deeper classification or rumen methanogenic taxa, was used to categorize the methanogenic ASVs according to defined clades (Seedorf et al., 2014).

After the generation of the ASV table, singletons/doubletons were removed and subsampling to an even depth of 4000 reads per sample was performed for all subsequent analyses. Alpha and Beta diversity analyses were performed and plotted using the PAleontological STatistics software (Hammer et al., 2001), including principal coordinate analysis (PCOA) using the Bray–Curtis dissimilarity metric and ASV richness, evenness, and Shannon index. Analysis of similarity (ANOSIM) was used to test the significance of the group clustering.

Centered log‐ratio transformation was performed for the statistical analysis of compositional data, using the ‘compositions’ R package (van den Boogaart & Tolosana‐Delgado, 2008). For most of the statistical analyses pertaining to the comparison between FL and protozoa‐associated prokaryotic, we used paired Wilcoxon tests unless otherwise stated. For all the analyses, *p* values of <0.05 after false discovery rate correction via the Benjamini‐Hochberg procedure were considered significant, unless otherwise stated in the text or figure.

## RESULTS

### Abundance and stability of the protozoa community across host animal diets

**FIGURE 1:**
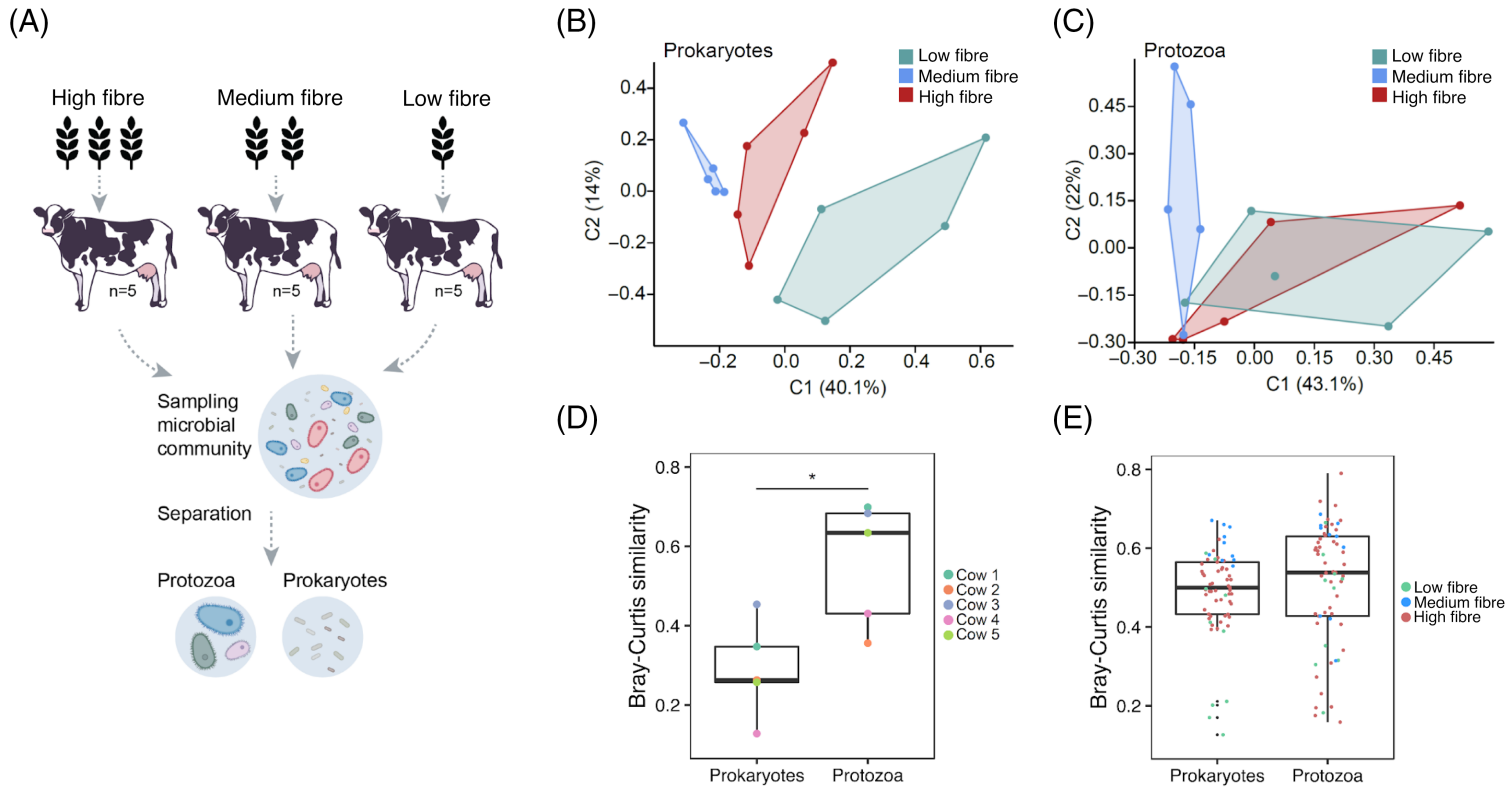
Comparison of the ecological dynamics of prokaryotes and protozoa across diets. (A) Scheme of the experimental set‐up used in this study. (B), (C) Principal coordinate analysis based on the Bray–Curtis pairwise similarity matrix for (B) prokaryotes and (C) ciliate protozoa. Each point represents a different sample plotted according to their amplicon sequence variant composition and abundance. A greater distance between two points infers a lower similarity in community compositions. The different colours represent the different animal diets (D) Bray–Curtis similarity between samples taken from the same five cows consecutively fed a different diet. Each point represents community similarity in each cow between the sample taken while fed high fibre and the sample taken while under low fibre diet, for the prokaryotic and protozoa community. Significance was obtained using Mann–Whitney *U* (*p* < 0.05). (E) Overall community similarity between samples from different cows (within diets low fibre; *n* = 5, medium fibre; *n* = 5, high fibre; *n* = 10) for the prokaryotic and protozoa community.

The diet of an animal encompasses a controllable determinant of changes in ruminal prokaryotic composition (Friedman et al., 2016; Jami et al., 2014; Mackie & McSweeney, 2020; Mizrahi & Jami, 2021; Newbold & Ramos‐Morales, 2020; Shaani et al., 2018). However, the effect of animal diet on the protozoa community and its associated prokaryotic community has seldom been investigated. Here we varied the animal feed by changing its fibre to concentrate ratios and followed the changes occurring in the protozoa community and their associated prokaryotes (Figure 1A). To this end three groups of five cows were allotted specific feeds for a minimum of 2 months before sampling, with one group referred to as HF receiving 80/20 fibre to grain ratio, MF with 50/50 ratio and LF receiving 30/70 ratio. After the habituation period, rumen fluid was sampled and used to characterize the abundance and composition of bacteria, archaea and protozoa. In order to characterize the protozoa‐associated prokaryotic community, a subset of the fresh samples was also used to separate the protozoa community from the FL prokaryotic community (Figure 1A).

Quantification of the abundance of ciliate protozoa across the different diets revealed that an increase in fibre content results in a decrease in the number of protozoa. This was observed when the analysis was performed using both quantitative PCR and live counting of the protozoa cells (Figure S1). The mean visual count for the HF (*n* = 5), MF (*n* = 5) and LF (*n* = 5) were 1.09 × 10^5^, 1.53 × 10^5^ and 2.91 × 10^5^ respectively. We did not find significant differences between archaea and bacterial abundances across the different diets (Figure S1).

We subsequently analysed the composition of the prokaryotic and protozoa community using 16S and 18S rRNA amplicon sequencing, respectively. In line with previous observations (Friedman et al., 2016; Jami et al., 2014; Mackie & McSweeney, 2020; Newbold & Ramos‐Morales, 2020; Shaani et al., 2018), the diet of the host animal had a pronounced effect on the prokaryotic community that was observed by a significant clustering of the samples based on diet using the Bray–Curtis pairwise dissimilarity metric (ANOSIM, *R* = 0.709, *p* < 0.001, Figure 1B). In contrast, the different diets had only a subtle effect on the protozoa community (ANOSIM, *R* = 0.185, *p* = 0.054 Figure 1C), and only showed a significant difference between the HF and MF groups (ANOSIM, *R* = 0.21, *p* = 0.034). No significant protozoa compositional difference was observed between the different diets due to the high variance observed across the samples within the same diet, although the genus *Dasytricha* tended to be more abundant as the fibre to concentrate ratio increased (Figure S2).

In order to test whether the protozoa community composition is more stable compared to the prokaryotic community when direct diet change occurs, the cows under an LF diet were switched to an HF diet, and sampled again after a 2 months adaptation period after the diet switch occurred. We monitored the degree of changes in microbial composition occurring within each individual animal. We observed that the protozoa community varied significantly less with dietary change compared to the prokaryotic community (Student's *t*‐test, *p* = 0.028, Figure 1C). The mean similarity within hosts in the prokaryotic community was 0.28 compared to 0.56 for the protozoa community, the latter not significantly different compared to the natural variation between cows within a given diet (Figure 1D). It is important to note that overall, the protozoa community was not more similar between samples (within the same diet) compared to the prokaryotic community (Figure 1E). This reduces the possibility that these results stem from a naturally more similar protozoa community due to the lower species richness of protozoa.

### Stability of the protozoa‐associated prokaryotic community across diets

**FIGURE 2:**
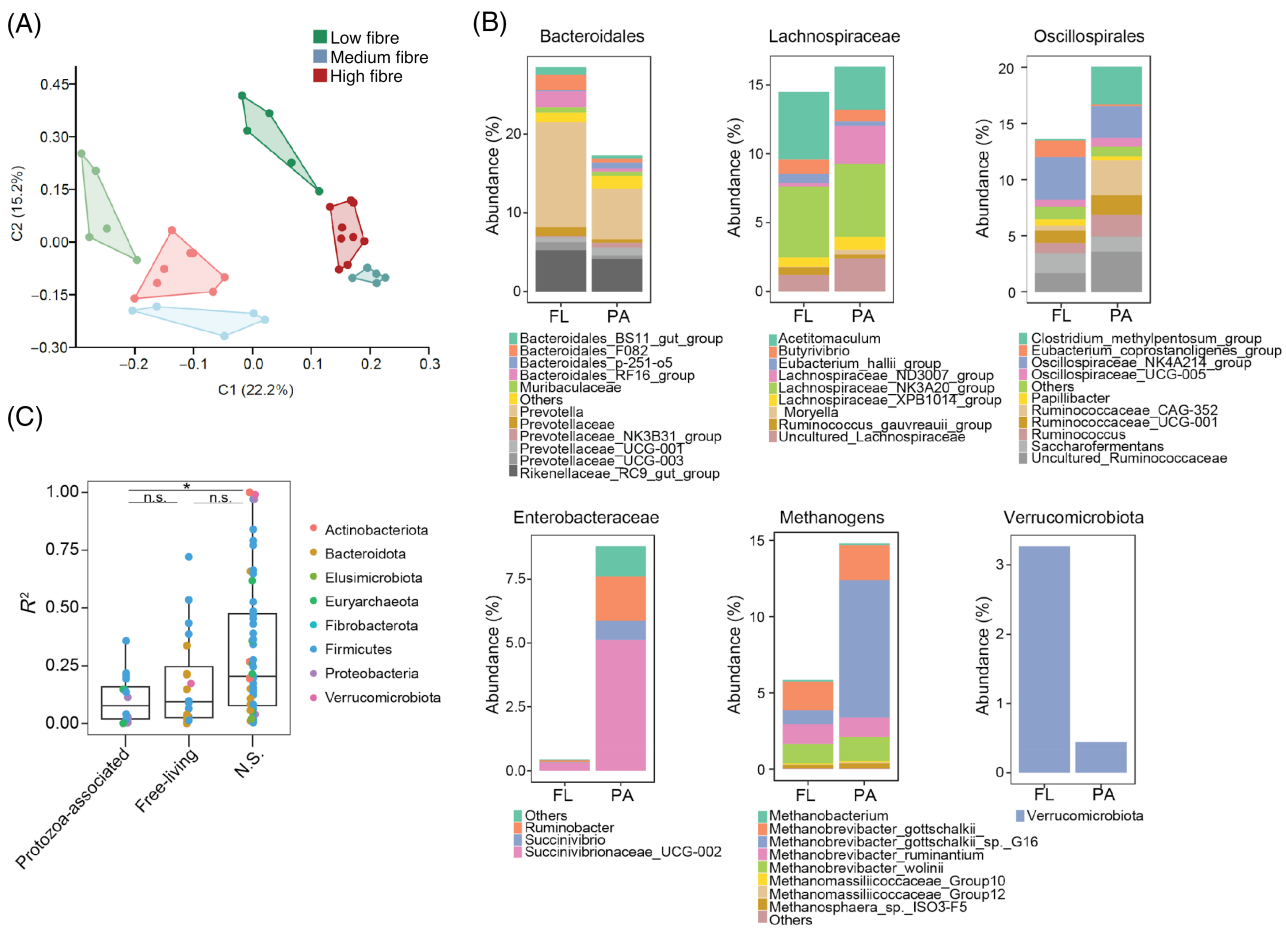
Compositional differences between the free‐living (FL) and protozoa‐associated (PA) prokaryotic community across diets. (A) Principal component analysis based on the pairwise similarity matrix obtained using the Bray–Curtis similarity index for the prokaryotic community. The different colours represent the different animal diets. The darker shades of each colour represent the FL community and lighter shades the PA community. (B) Community composition between the FL and PA prokaryotic community. Taxonomic classification was obtained using the Silva 13.8 16S rRNA database (Yilmaz et al., 2014). (C) Box plot denoting the distribution of coefficient of variation between the FL prokaryotic abundance and PA prokaryotic abundance. Significance between the boxes was assessed using Student's *t* test, with significance denoted by an asterisk (*p* < 0.05), and n.s denoting that no significance was found between the boxes.

As the prokaryotic community was different between the different diets, we sought to assess whether this change in composition is also reflected in the protozoa‐associated community. The higher stability of the protozoa community across diets led us to hypothesize that the prokaryotic community associated with protozoa may serve as a reservoir for taxa thereby remaining present in the rumen across differing conditions. To characterize the prokaryotic community that is physically associated with protozoa, the protozoa community was separated from the FL community in the samples taken from cows under the different diets (Figure 1A). The samples were also separated into sub‐communities in order to further assess whether different protozoa taxa carry different prokaryotic communities. Our results showed that the prokaryotic community associated with protozoa (protozoa associated = PA) exhibits corresponding, significant differences in composition driven by different diets based on the Bray–Curtis dissimilarity metric (ANOSIM, *R* = 0.615, *p* = 0.001, Figure 2A, Table S2). However, the communities also showed discrimination between the protozoa‐associated community and the FL community (ANOSIM, *R* = 0.57, *p* = 0.0001, Figure 2A, Table S2). There were no observable differences between the prokaryotic sub‐communities stemming from different protozoa sub‐communities (Figure S3a).

Thus, while diet imposes an effect on the prokaryotic community structure associated with protozoa, the community composition remains unique to the protozoa association.

### Protozoa‐associated community is enriched for specific taxa regardless of diet

Our results so far showed that while the prokaryotic community associated with protozoa is affected by the dietary change, it still diverges significantly from the FL community. We therefore hypothesize that the divergence is a result of key taxa that adapted to the protozoa microenvironment.

At the phylum level, the protozoa‐associated community was significantly enriched in *α* *and γ‐Proteobacteria* (FL = 0.03% vs. PA = 0.42% and FL = 0.44% vs. PA = 7.9%, respectively), and Euryarchaeota (FL = 5.4% vs. PA = 14.9%), while Bacteroidetes and Verrucomicrobia were significantly more prevalent in the FL communities (FDR corrected paired Wilcoxon, *p* < 0.01; Figure 2B, Table S3). Further characterization of the differentially abundant taxa among the protozoa‐associated communities revealed that *Ruminococcaceae_CAG‐352* (FL; 0.46% vs. PA; 3.1%) and Clostridiales [methylpentosome] (FL; 0.09% vs. PA; 3.3%) belonging to *Oscillospiraceae* (Bacillota) and *Lachnospiraceae* ND_3007 (FL; 0.24% vs. PA; 3.9%) from Lachnospirales were significantly enriched among the protozoa‐associated population (FDR corrected Paired Wilcoxon *p* < 0.01; Figure 2B, Figure S3b,c, Table S3). From the *γ‐Proteobacteria* class, *Succinivibrionaceae*, *Ruminobacter* and *Succinivibrionaceae* UCG‐002 were all significantly enriched in the protozoa‐associated prokaryotic community. The protozoa‐associated communities were also shown to uniquely harbour a narrow number of ASVs belonging to the taxa mentioned such as ASVs belonging to *Methanobrevibacter*, *Succinovibrionaceae*, Clostridiales [methylpentosome] and Rickettsiales. Within the *Methanobrevibacter* genus, there were several ASVs detected, and associated with *Methanobrevibacter gottschalkii* sp.16 based on the RIM‐DB (Seedorf et al., 2014) that were exclusive to the protozoa‐associated communities and those represented the majority of the methanogens found (Figure 2B, Table S3). Thus, the protozoa community harbours a subset of prokaryotic taxa that are unique or enriched regardless of the environmental conditions and the composition of the FL community prevailing.

In order to further determine the independent nature of the enriched taxa found associated with protozoa we assessed the coefficient of determination using linear regression obtained between the abundance of the enriched taxa associated with protozoa and their relative abundance in the FL fraction. This determines, in addition to the enrichment, whether the relative abundance found is a direct result of the change in abundance in the FL community (between diets for instance). This analysis showed that the *R* ^2^ of significantly enriched prokaryotic ASVs in protozoa was significantly lower than those who were not significantly associated with protozoa, suggesting that the enrichment of the specific taxa enriched in the protozoa‐associated community is independent of the relative abundance in the FL community (Figure 2C). Our results thus show that a subset of taxa preferentially associates with the protozoa community and this association is stable across diets.

### The protozoa‐associated prokaryotic community is enriched in genes belonging to hydrogenotrophic pathways

Similar to previous studies our results reveal the association between protozoa and the methanogen community (Figure 2B) (Levy & Jami, 2018; Lloyd et al., 1996). This association is suggested to be the result of the release of hydrogen by the protozoa and its direct consumption by the hydrogenotrophic methanogens to produce methane (Newbold et al., 2015; Ushida et al., 1997). We thus hypothesize that hydrogen may be a strong determinant of microbial associations with protozoa and that protozoa may encompass a hub for diverse hydrogenotrophic functions beyond those observed for methanogens (Greening et al., 2019; Li et al., 2022; Morgavi et al., 2010). Interestingly, we observed that taxa such as *Oscillospiraceae* and *Lachnospiraceae* were significantly enriched in the protozoa‐associated prokaryotic community, which are taxa that have been previously shown to utilize hydrogen for reductive acetogenesis and the Wood Ljungdahl pathway (Gagen et al., 2010, 2014, 2015).

**FIGURE 3:**
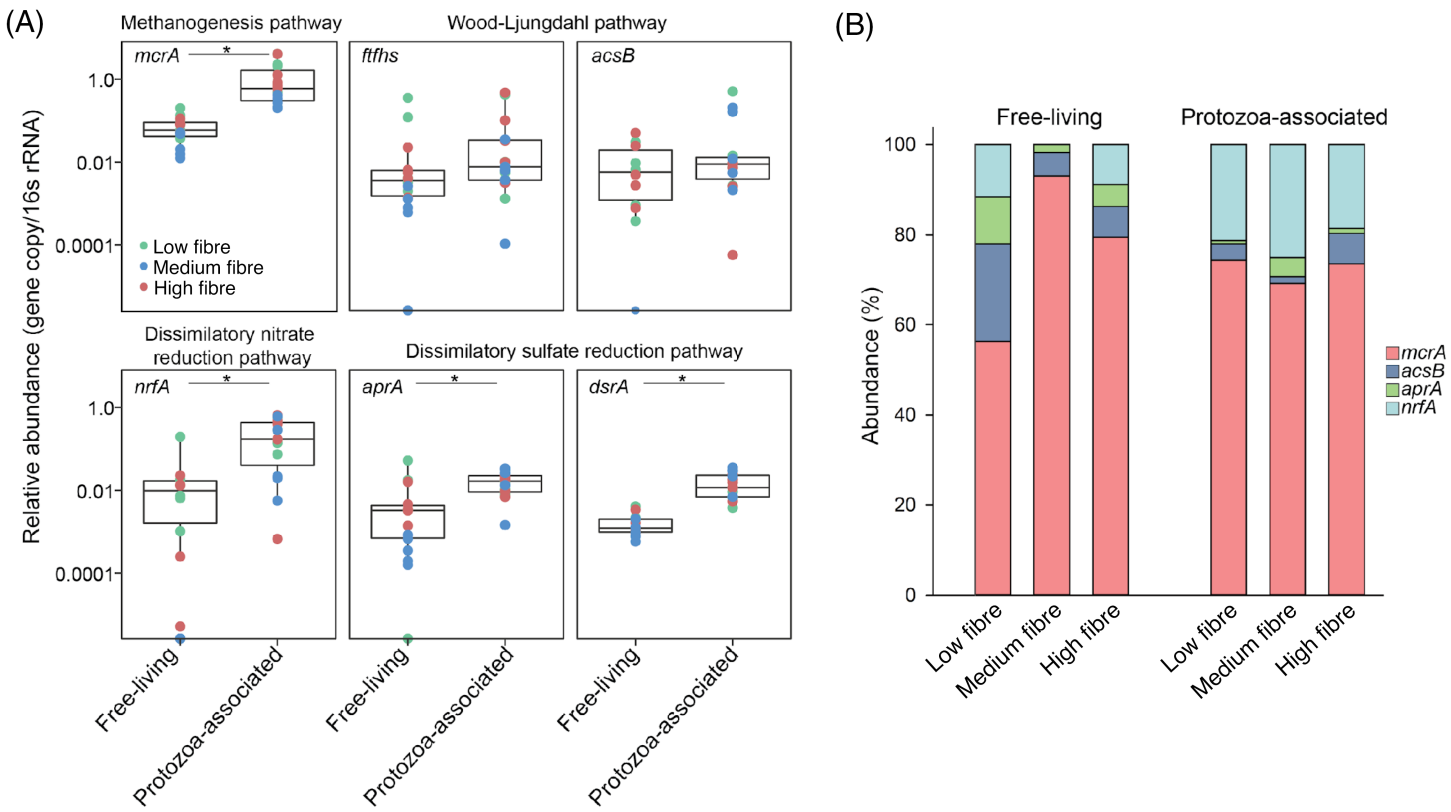
Relative abundance of key genes in hydrogenotrophic pathways in the free‐living and protozoa‐associated prokaryotic community. (A) Box plots illustrating the abundance of genes belonging to known hydrogenotrophic pathways in the rumen in both free‐living and protozoa‐associated prokaryotic communities of the rumen. Significance between the groups, denoted by an asterisk “*”, was assessed using a paired Wilcoxon statistical test with false discovery rate correction (*p* < 0.05). (B) Proportion of genes representing these pathways observed across various diets within the free‐living and protozoa‐associated prokaryotic communities of the rumen.

In order to test whether hydrogenotrophic functions are enriched associated with protozoa, we investigated the presence of other bacterial reductive pathways known to be prevalent in the rumen. To do this we quantified key genes of pathways related to hydrogen utilization and reduction of end molecules known to be present in the rumen and evaluated their abundance in the protozoa‐associated community compared to the FL community. Our results showed that the *mcrA* gene was the most abundant gene in our samples and was significantly higher in the protozoa‐associated communities compared to the FL community (Figure 3A,B), which is in line with the higher proportion of methanogens reads observed using the amplicon sequencing (Figure 2B). The *ftfhs* and *acsB* genes, belonging to the Wood Ljungdahl pathway did not exhibit a significant enrichment in the protozoa‐associated prokaryotic community compared to the FL community (Figure 3A). The *nrfA* gene of the dissimilatory nitrate reduction pathway, which was generally the second most abundant gene in the samples, was significantly enriched in protozoa‐associated bacterial communities reaching up to 10‐fold higher abundance compared to the FL community (paired Wilcoxon; *p* < 0.01, Figure 3A). Such enrichment was also seen in the genes *aprA* and *dsrA* belonging to the dissimilatory sulfate reduction pathway (paired Wilcoxon; *p* < 0.05, Figure 3A). When assessing the proportion for the genes of each pathway by summing up the overall gene copies for each gene together (with *acsB* and *aprA* being representatives of their respective pathways), we find that overall the *nrfA* gene exhibited a significantly higher proportion in the protozoa‐associated community (21.6%) compared to the FL community (6.8%), regardless of the diet (paired Wilcoxon; *p* < 0.05, Figure 3B). Our results overall show that specific bacterial hydrogenotrophic activity may be higher among the protozoa‐associated prokaryotic community compared to the FL community supporting hydrogen as a potential driver of microbial association between protozoa and prokaryotes beyond methanogenesis.

### Phylogenetic assignment of the hydrogen utilizing functions

**FIGURE 4:**
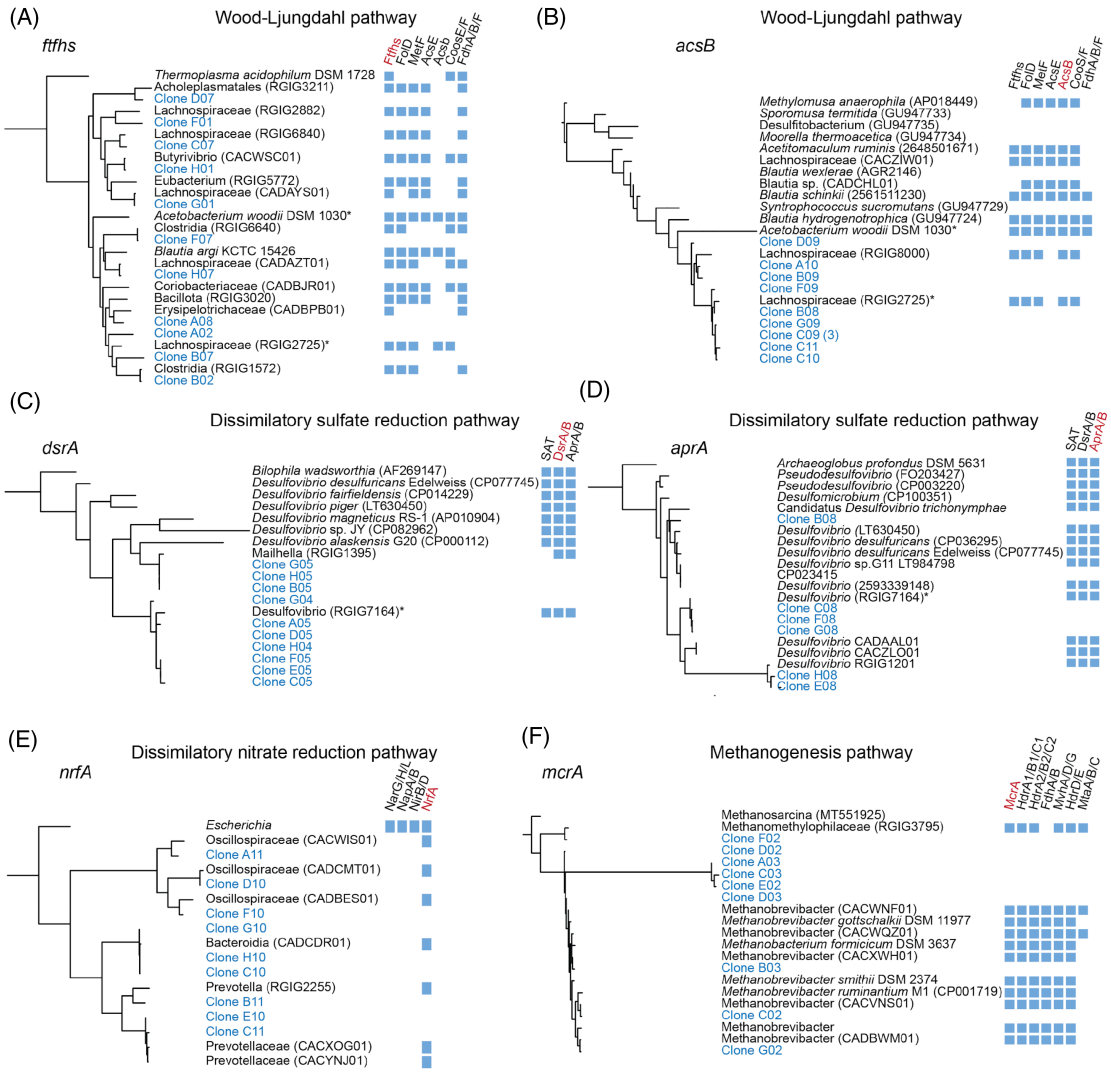
Phylogenetic assignment of the gene sequences and complementarity of hydrogenotrophic pathways. Phylogenetic trees of gene sequences obtained in this study (in blue), metagenome‐assembled genomes (MAGs) obtained from previous large‐scale metagenomic analysis (accessions starting with ‘CA’ or ‘RGIG’; Stewart et al., 2019; Xie et al., 2021), and sequences from the NCBI database. The genomes of homologues were searched against genes of the Wood‐Ljungdahl pathway (A, B), the dissimilatory sulfate reduction pathway (C, D), the dissimilatory nitrate reduction pathway (E) and the hydrogenotrophic methanogenesis pathway (F). The presence of specific genes is indicated by blue squares. Species or MAGs that are shared between genes from the same pathway are marked with an asterisk.

Our results show the presence and stability of specific prokaryotic taxa with the protozoa environment (Figure 2) as well as an enrichment in specific hydrogenotrophic functions (Figure 3). In order to integrate the specific taxa with the enrichment in functions, the amplified gene sequences from the different hydrogenotrophic pathways were cloned and sequenced. The gene sequences were then compared to curated metagenome assembled genomes (MAGs) datasets obtained from large‐scale surveys of the rumen microbiome (Stewart et al., 2019; Xie et al., 2021), alongside previous sequencing efforts obtained for the different hydrogenotrophic functions in the rumen and bacterial isolates known to carry the pathways (Gagen et al., 2010, 2015, Figure 4A–F). Out of eleven sequences obtained from the *ftfhs* gene, 10 had a closest match to Clostridiales MAGs, and more specifically, to *Lachnospiraceae*, with sequence similarities ranging between 69% and 99% to their closest relative (Figure 4A, Table S4). This result also matches the enrichment of these taxa observed through the 16S rRNA analysis (Figure 2B). We further assessed whether the full Wood‐Ljungdahl pathway was present in the closest related MAGs to our sequences (Figure 4). For the case of the *ftfhs* gene, only one of the sequences obtained from the protozoa‐associated gene amplification was similar to a MAG carrying both the *ftfhs* and the *acsB* genes (Figure 4A). The *acsB* sequences in contrast were associated with MAGs carrying both genes and had generally a more complete Wood‐Ljungdahl pathway (Figure 4B). Notably, only one *Lachnospiraceae* MAG associated with many sequences from *ftfhs* and *acsB* genes could be found. This strengthens previous observations that *acsB* may constitute a better marker for the assessment of reductive acetogenesis (Carbonero et al., 2012; Gagen et al., 2015).

All the *dsrA* and *aprA* genes from the dissimilatory sulfate reduction pathway were attributed to MAGs of the *Desufovibrionaceae* family, with a shared *Desulfovibrio* MAG (Figure 4C,D). Interestingly, although not identified by the original statistical analysis of the 16S rRNA data (which considered only ASVs and genera exhibiting an average relative abundance higher than 0.5% in at least one group), this family was in a significantly higher relative abundance in association with protozoa (Figure S4). For the ammonia‐forming nitrite reductases (*nrfA*), the gene sequences matched MAGs belonging to *Prevotellaceae* (6 clones) and *Oscillospiraceae* (4 clones) and were the only gene present from the dissimilatory nitrate reduction pathway in the closely related MAG sequences.

## DISCUSSION

The ecological dynamics of ciliate protozoa in the rumen remains seldom analysed when compared to bacteria and archaea. This phenomenon is not only true for the rumen environment but has been true for most other environments leaving a gap in our understanding of complex microbial communities (Caron et al., 2009; Gao et al., 2019; Solomon & Jami, 2021). Our results show that while the rumen prokaryotic community responds strongly to changes in fibre content in the diet as a community, the protozoa do not respond to changes in their environment caused by the differing diets. More specifically, while the protozoa population size increases with decreased fibre content in the feed as previously observed (Hook et al., 2011; Towne et al., 1988), there were no significant changes in community composition, as opposed to the bacterial community. Henderson et al, which assessed the protozoa community across different cohorts of different species of ruminants, similarly reported a strong host individuality in protozoa communities even within specific cohorts (Henderson et al., 2015). Furthermore, a recent study in the yak rumen found several differences in the relative abundance of some protozoa species in response to feed type, but overall community structure as measured by Beta diversity was not significantly different as opposed to bacterial or the fungal community (Cui et al., 2022).

Our results further reveal a higher stability of protozoa species compared to prokaryotic species in the rumen, when examining the community changes under varying diets (Figure 1C). This observation was in line with previous results showing that changes in fibre to concentrate ratio significantly affected bacterial, archaeal and fungi composition, but not the protozoa community structure (Tapio et al., 2017). In contrast, supplementation of the animal feed with lipids was shown to have an effect on the protozoa community and protein expression (Andersen et al., 2023; Tapio et al., 2017). Interestingly, the study by Andersen et al. (2023) also noted that when a high starch diet is given, protozoa species with well‐established starch degradation functions, are not necessarily more active, which they propose is the result of competition with starch‐degrading bacteria or sub‐optimal pH conditions in the rumen.

The large difference in the response of protozoa and prokaryotes to stark changes in the rumen environment such as animal diet hints to diverse unexplored ecological forces driving the community of protozoa in the rumen. The individuality in host animals may be driven by genetic factors (Wallace et al., 2019), or alternatively, differential early acquisition may dictate protozoa composition later in life. The latter alternative may carry important implications as to the possibility of early intervention in order to stably modulate the protozoa community (Mizrahi & Jami, 2021).

Studies on association between protists and prokaryotes in general and specifically ciliate protozoa have shown that the nature of interaction can be diverse, ranging from antagonistic interaction such as predation, to commensal, all the way to mutualistic interactions (Gao et al., 2019; Gast et al., 2009; Paisie et al., 2014; Pernthaler, 2005).

Our results indicate that the ciliate protozoa‐associated prokaryotic community is strongly driven by the community surrounding them, and changes induced in this community result in different associated communities. This was seen on two levels, with different diets modulating the protozoa‐associated bacterial community and on the individual level, as the composition reflected the FL community in each individual host. However, our results also show that the protozoa‐associated community retains significant differences when compared to the FL community regardless of diet, suggesting that a certain degree of selection may occur in this community. This is particularly evident for taxa belonging to the Proteobacteria phylum, methanogens and Oscillospirales. The differences observed could stem from preferential predation for which inconclusive evidence exists for rumen protozoa (Coleman, 1964; de la Fuente et al., 2011; Gutierrez, 1958), or from the provision of habitats and nutrients for mutualistic exchange with their associated bacteria.

It is notable that none of the hydrogen‐utilizing gene sequences obtained belonged to the *γ‐Proteobacteria* phylum, despite it being highly enriched in the protozoa‐associated community. Furthermore, in a previous study, *γ‐Proteobacteria* were also highly enriched in the presence of protozoa in in‐vitro rumen microcosms compared to microcosms incubated without protozoa (Solomon et al., 2022). As these taxa were shown to carry genes conferring them resistance to predation (Gong et al., 2016), their presence may reflect their persistence and accumulation in the prokaryotic community associated with protozoa.

As well‐established hydrogen‐producers, protozoa are suggested to create an attractive micro‐environment for taxa such as methanogens that utilizes hydrogen to reduce CO_2_ to methane (Newbold et al., 2015; Ushida et al., 1997). By quantifying key genes belonging to hydrogen utilizing pathways, other than methanogenesis, known to exist in the rumen, we found that the community associated with ciliate protozoa is significantly enriched in those genes. In the case of potential nitrite and sulfate‐reducing taxa, the gene sequences obtained could be attributed to taxonomic affiliations seen as enriched in association with protozoa via amplicon sequencing. Our results thus suggest that ciliate protozoa in the rumen may be a hub for various hydrogenotrophic functions, beyond methanogenesis. This observation is not without precedents and different types of hydrogen transfer‐based putative mutualism have been suggested between prokaryotes and ciliate (Bernhard et al., 2000; Gast et al., 2009; Ott et al., 2004). In termites, putative bacterial symbionts from the Myxococcota (formerly *Deltaproteobacteria*) and Spirochaetota phylum were shown to have a wide range of hydrogenotrophic function which appear to be the nature of the interaction between them and their flagellate protozoa host (Ikeda‐Ohtsubo et al., 2016; Kuwahara et al., 2017; Ohkuma et al., 2015). Likewise, in anoxic fresh water lake, a ciliate endosymbiont was found to supply its ciliate host with energy via denitrification performed by hydrogen transfer (Graf et al., 2021). In the rumen, metabolic interactions between ciliate protozoa and nitrate/nitrite‐reducing bacteria have been proposed (Lin et al., 2011; Roman‐Garcia et al., 2019; Villar et al., 2020; Welty et al., 2019). When co‐cultured with bacteria, rumen protozoa were reported to accelerate nitrate reduction (Villar et al., 2020), and the protozoal fraction, which likely included both protozoa‐associated prokaryotes showed a greater ability to reduce nitrate without accumulation of nitrite (Lin et al., 2011), than the prokaryotic community alone. The possibility that, combined nitrate reduction to nitrite by protozoa, for which genomic evidence exists, with production of hydrogen may lead to the enrichment of nitrite‐reducing bacteria (Roman‐Garcia et al., 2019; Welty et al., 2019). Considering the observation that exogenous addition of nitrite (but not nitrate) severely inhibits motility and chemotaxis of protozoa (Roman‐Garcia et al., 2019), we can speculate that its clearance by associated bacteria might encompass the mechanism of mutualistic interaction between them. Our results suggest that the protozoa‐associated microbiome may possibly complete the reduction of nitrate. Successful decoupling of protozoa and associated bacteria would be required to assess such. As nitrate and other nitrogenous compounds are being evaluated for their potency to reduce methane, the role of protozoa and their associated microbes in metabolizing these compounds and their contribution to the mitigation of methane is an interesting avenue for further research (Morgavi et al., 2023; Yang et al., 2016).

## CONCLUSION

Our study sheds light on the underexplored ecological dynamics of ciliate protozoa in the rumen, emphasizing the significant disparity in their response to environmental changes, in this case, dietary shifts, when compared to prokaryotic communities. While the prokaryotic community exhibits a strong collective response to alterations in diet, the protozoa population size is influenced by dietary changes but shows no significant shifts in community composition. This observation underscores the stability of protozoa in the rumen and hints at diverse, unexplored ecological forces governing their community dynamics.

Our findings reveal that although the ciliate protozoa‐associated prokaryotic community is largely defined by their surrounding prokaryotic community, the retention of significant differences in the protozoa‐associated community, irrespective of diet, suggests a level of selection occurring within this community, which may be the result of mutualistic interaction or selective predation. Furthermore, our investigation into key hydrogen‐utilizing pathways and associated genes indicates that ciliate protozoa in the rumen may serve as a hub for various hydrogenotrophic functions, extending beyond methanogenesis. The potential metabolic interactions between protozoa and nitrate/nitrite‐reducing bacteria suggest a complex relationship that could contribute to nitrate reduction and may have implications for methane mitigation strategies. Overall, our results reveal the potentially impactful role of ciliate rumen protozoa as stable interaction partners and as a source of energy and habitat for diverse prokaryotic species. As we unveil the intricate dynamics of protozoa in the rumen, further research into their ecological roles and interactions holds promise for advancing our understanding of microbial communities and their potential contributions to addressing environmental challenges, such as methane reduction.

## AUTHOR CONTRIBUTIONS

**Elie Jami:** Conceptualization; investigation; writing – original draft; funding acquisition. **Ido Toyber:** Conceptualization; investigation. **Raghawendra Kumar:** Investigation; writing – original draft; writing – review and editing.

## CONFLICT OF INTEREST STATEMENT

The authors declare no conflicts of interest.

## Supporting information


**Figure S1.** Abundance of prokaryotes and protozoa across animal diets.
**Figure S2.** Protozoa composition across diets.
**Figure S3.** Prokaryotic composition of the protozoa associated community.
**Figure S4.** Abundance of *Desulfovibrionaceae*.
**Table S1.** Primers used for community analysis, amplicon sequencing, and hydrogen utilization.
**Table S2.** ANOSIM analysis across the different prokaryotic communities and across the different diets.

**Table S3.** Genus abundances.

**Table S4.** Gene similarities.

## Data Availability

The sequencing data used for this study are openly available in the NCBI SRA database under BioProject ID PRJNA1067207 and PRJNA1065150.
