# Supplementary material for: Rumen protozoa are a hub for diverse hydrogenotrophic functions
Source: Environ Microbiol Rep. 2024 Jul 3;16(4):e13298. doi: 10.1111/1758-2229.13298 (PMC11222294; doi:10.1111/1758-2229.13298)
Supplement: Supplementary file 1 — Figure S1. Abundance of prokaryotes and protozoa across animal diets. Figure S2. Protozoa composition across diets. Figure S3. Prokaryotic composition of the protozoa associated community. Figure S4. Abundance of Desulfovibrionaceae. Table S1. Primers used for community analysis, amplicon sequencing, and hydrogen utilization. Table S2. ANOSIM analysis across the different prokaryotic communities and across the different diets. [file EMI4-16-e13298-s002.pdf]

Rumen protozoa are a hub for diverse hydrogenotrophic functions

Ido Toyber[1,2*], Raghawendra Kumar[1*], Elie Jami[1#]

[1]Department of Ruminant Science, Institute of Animal Sciences, Agricultural Research Organization, Volcani Center, Rishon LeZion, Israel
[2]Department of Animal Science, the Hebrew University of Jerusalem, Rehovot 7610001, Israel

*These authors contributed equally to this work
#Correspondance: elie@volcani.agri.gov.il

Supplementary material

Figure S1. Abundance of prokaryotes and protozoa across animal diets.

Figure S2. Protozoa composition across diets.

Figure S3. Prokaryotic composition of the protozoa associated community.

Figure S4. Abundance of *Desulfovibrionaceae*.

Table S1. Primers used for community analysis, amplicon sequencing, and hydrogen utilization.
Table S2. ANOSIM analysis across the different prokaryotic communities and across the different diets.

Table S3. Genus abundances.

Table S4. Gene similarities.

Supplementary figures

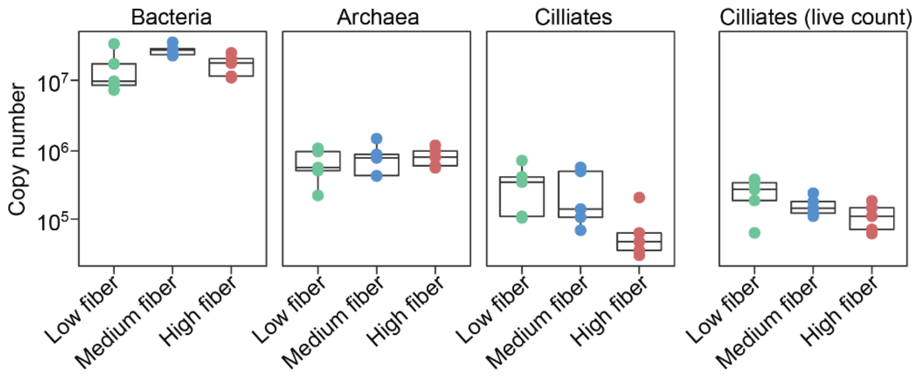

Figure S1. Abundance of prokaryotes and protozoa across animal diets. The abundance of bacteria, archaea and ciliate protozoa across different diets was quantified using quantitative PCR with the appropriate primers (see methods). The plot on the right shows the number of ciliate protozoa evaluated by counting protozoa cells under a microscope (see methods).

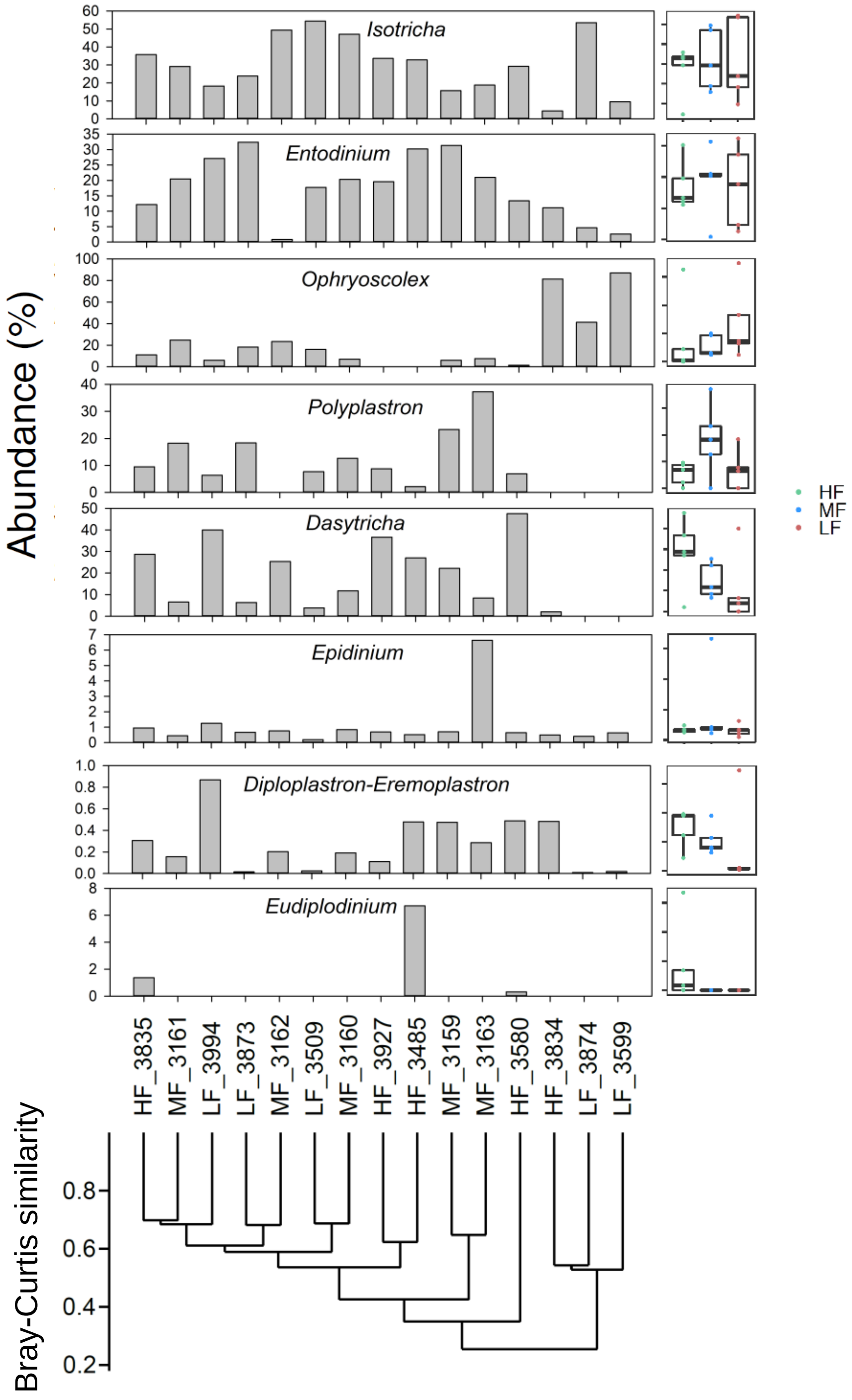

Figure S2. Protozoa composition across diets. Genus level protozoa abundance across each individual cow (right bar panels) and average abundance for each genus across the different diets (left box plot panels). The bottom panel represents the UPGMA dendrogram obtained by the pairwise similarity matrix based on the Bray-Curtis metric.

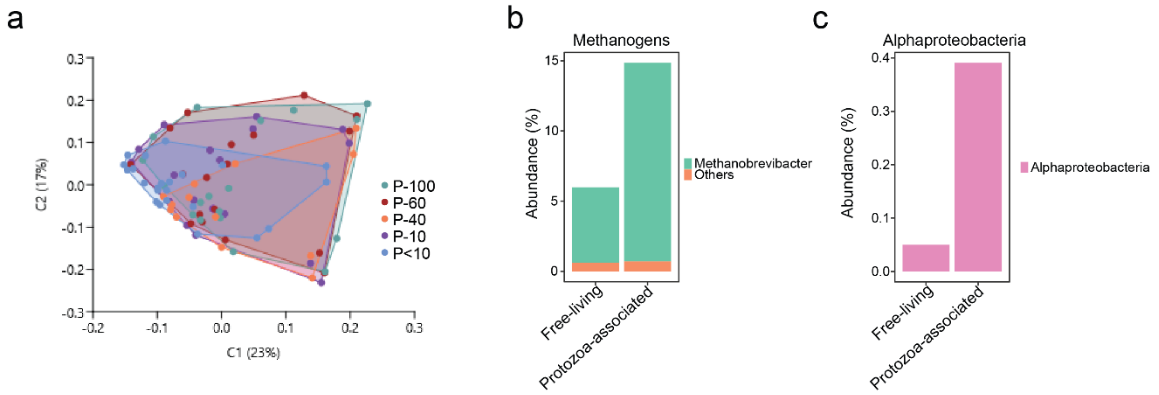

Figure S3. Prokaryotic composition of the protozoa associated community. (a) Principal coordinate analysis (PcoA) between different sub-communities of protozoa within each cow. (b), (c) Relative abundance of Methanogens and Alphaproteobacteria in the free-living community vs. the protozoa associated community.

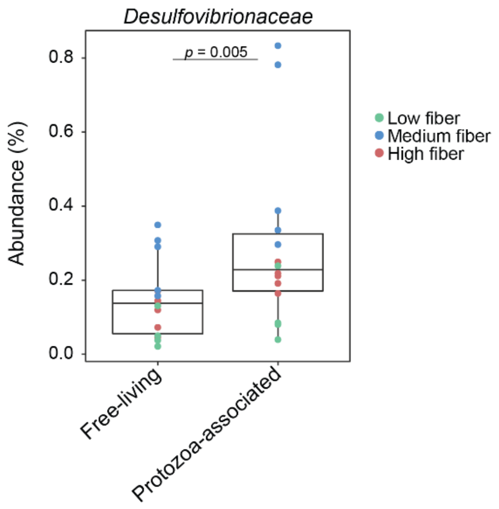

Figure S4. Abundance of *Desulfovibrionaceae*. The abundance of Desulfovibrionaceae was evaluated using 16s rRNA sequencing for free-living bacteria and protozoa-associated bacteria across the different diets. Paired Wilcoxon test was used to assess significance.

Table S1. Primers used for community analysis, amplicon sequencing, and hydrogen utilization

Primer name	5'-3' sequence	Size (bp)	Target	Reference
mcrA F	TTCGGTGGATCDCARAGRGC	140	Archaeal methyl coenzyme-M reductase	Denman et al. (2007)
mcrA R	GBARGTCGWAWCCGTAGAATCC			
ftfhs F	TTYACWGGHGAYTTCCATGC	1102	Formyltetrahydrofolate Synthetase Gene (Wood-Ljungdahl pathway)	Gagen et al. (2010)
ftfhs R	GTATTGDGTYTTRGCCATACA			
nrfA F	ATGACCCGGATACCATG	498	Nitrite reductase (Dissimilatory nitrate reduction)	Asanuma et al. (2015)
nrfA R	AGTGGGCTGGTCATCCA			
acsB F	CTYTGYCAGTCMTTYGCBCC	416	acetyl-CoA synthase (Wood-Ljungdahl pathway)	Gagen et al. (2010)
acsB R	CCCATAAABCCYGGDGTYTG			
aprA F	TGGCAGATMATGATYMACGGG	396	adenosine-5′-phosphosulfate reductase alpha subunit (aprA) Sulfate Reducing	Deplancke et al. (2000)
aprA R	GGGCCGTAACCGTCCTTGAA			
dsrA F	CCAACATGCACGGYTCCA	162	dissimilatory sulfite reductase alpha subunit	Devkota et al. (2012)
dsrA R	CGTCGAACTTGAACTTGAACTTGTAGG			

Table S2. ANOSIM analysis across the different prokaryotic communities and across the different diets. The values on the lower left side of the table represent the R value denoting the difference between the different group (closer to 1 = more dissimilar) and the upper right side of the table the corresponding p-values.

R/P	Free-living HF	Free-living LF	Free-living MF	Protozoa associated HF	Protozoa associated LF	Protozoa associated MF
Free-living HF		0.0015	0.843	0.0015	0.006	0.006
Free-living LF	0.7418		0.12	0.0165	0.09	0.09
Free-living MF	0.2669	0.84		0.006	0.132	0.09
Protozoa associated HF	0.8447	0.887	0.8831		0.0345	0.1935
Protozoa associated LF	0.9535	0.824	0.932	0.5623		0.04
Protozoa associated MF	0.9367	0.964	0.864	0.3478	0.592	

Table S3. Genus abundances. Relative abundance of prokaryotic genera in the free-living and protozoa associated community and under the different diets. Significance was obtained on the CLR transformed data using paired FDR corrected Wilcoxon test with the threshold value for significance at $p < 0.05$.

Table S4. Gene similarities. Similarity of clone sequences from this study to closest homolog in databases (Figure 4; see methods).
